# Dietary Assessment by Pattern Recognition: a Comparative Analysis

**DOI:** 10.1016/j.cdnut.2023.101999

**Published:** 2023-09-11

**Authors:** Adam M. Bernstein, Lauren Q. Rhee, Valentine Y. Njike, David L. Katz

**Affiliations:** 1 Diet ID, Inc., Detroit, MI, USA; 2 Yale-Griffin Prevention Research Center, Griffin Hospital, Derby, CT, USA

**Keywords:** dietary assessment, diet quality photo navigation, pattern recognition, diet quality, nutrition

## Abstract

**Background:**

Diet quality photo navigation (DQPN) is a novel dietary intake assessment tool that was developed to help address limitations of traditional tools and to easily integrate into health care delivery systems. Prevailing practice is to validate new tools against approaches that are in wide use.

**Objective:**

This study aimed to assess *1*) the validity of Diet ID in measuring diet quality, food group and nutrient intake against 2 traditional dietary assessment methods (i.e., food record [FR], food frequency questionnaire) and *2*) the test reproducibility/reliability of Diet ID to obtain similar results with repeat assessments.

**Methods:**

Using a participant-sourcing platform for online research, we recruited 90 participants, 58 of whom completed DQPN, a 3-d FR (via the Automated Self-Administered 24-hour Dietary Assessment Tool), and a food frequency questionnaire (FFQ, via the Dietary History Questionnaire III). We estimated mean nutrient and food group intake with all 3 instruments and generated Pearson correlations between them.

**Results:**

Mean age (SD) of participants was 38 (11) y, and more than half were male (64%). The strongest correlations for DQPN when compared with the other 2 instruments were for diet quality, as measured by the Healthy Eating Index 2015; between DQPN and the FFQ, the correlation was 0.58 (*P* < 0.001), and between DQPN and the FR, the correlation was 0.56 (*P* < 0.001). Selected nutrients and food groups also showed moderate strength correlations. Test-retest reproducibility for measuring diet quality was evaluated for DQPN and showed a correlation of 0.70 (*P* < 0.0001).

**Conclusions:**

The current study offers evidence that DQPN is comparable to traditional dietary assessment tools for estimating overall diet quality. This performance, plus DQPN’s ease-of-use and scalability, may recommend it in efforts to make dietary assessment a universal part of clinical care.

## Introduction

Poor diet quality is a leading risk factor for premature death and disability [[1], [2], [3]], and public health authorities have called for new approaches to integrate nutrition into health care delivery [4]. The American Heart Association has stated that dietary intake should be captured in every patient record and addressed routinely in clinical care [5]. Traditional dietary assessment methods, however, have well-recognized limitations, including that they are memory-dependent, time-consuming, and difficult to scale [6,7].

Diet quality photo navigation (DQPN) was developed to help address limitations of traditional dietary assessments and for easy integration into health care delivery systems. It is a patented, digital application, leveraging a fundamentally new approach to dietary assessment, and currently marketed solely by Diet ID (www.dietid.com) [8,9]. The method uses pattern recognition, a universal human aptitude rooted in evolutionary biology [10], rather than the recollection of food intake, a known human frailty [6,11,12]. It can be completed within minutes through a variety of digital delivery systems and is not limited by low literacy or numeracy.

Multiple studies to date have reported on the performance of DQPN, comparing the novel assessment with 24-h recalls, skin carotenoids, and cardiometabolic biomarkers [7,8,13,14]. The ideal validation would be against an entirely reliable gold standard. For dietary intake assessments, that is generally considered to be metabolic ward studies. Such studies are often precluded by cost, convenience, and scalability. As a result, prevailing practice is to validate new approaches against the approaches currently in wide use, including 24-h recalls, food records (FRs), and food frequency questionnaires (FFQs) [15].

The performance of DQPN relative to established methods is timely and critical given both the growing interest in food-as-medicine and the importance of measuring diet quality at scale in the service of managing it. To that end, we conducted a study to evaluate the validity of the DQPN. The objective was to assess the validity of Diet ID in measuring diet quality, food group, and nutrient intake compared with a FFQ and multiple FRs. We hypothesized that DQPN would correlate well with the other instruments for diet quality. A secondary aim was to examine the test-retest reliability of DQPN.

## Methods

### Research platform and study population

The study was conducted by using CloudResearch (www.cloudresearch.com), a participant-sourcing platform for online research which has access to the Amazon Mechanical Turk (MTurk) participant panel (www.mturk.com). CloudResearch recruited from their Approved Participant list (i.e., high quality subsample of the MTurk population), a study population of US adult volunteers without regard to sex, race, ethnicity, or any other defining characteristics. Study inclusion criteria were that participants must be able to commit to the required tasks and time frame and agree not to change their diet during the course of the study. Participants were excluded if they had changed their dietary pattern significantly within the preceding 12 mo or followed a specialized diet (e.g., liquid diet or restrictive medically prescribed diet).

Sample size estimates were determined as follows: correlation coefficients between FFQs and FRs from validation studies have been reported in the range of 0.3 to 0.6 for most nutrients (and in the range of 0.4 to 0.8 after statistical adjustment [deattenuation] for energy intake and within-person variation), with the mean clustering around 0.5 [16]. The correlation coefficient between a FFQ and repeat FFQs (from reliability studies) have been in the range of 0.4 to 0.7 [16]. For the proposed study, a correlation coefficient of 0.4, significance of 0.05, and power of 0.8 would require at least 47 participants. We aimed for a sample size of 60 participants, and 90 adult participants were targeted for participation to allow for attrition.

Participants were compensated for each completed dietary assessment, described below, according to rates set by CloudResearch and MTurk policies, in such a way that would not result in more than a total of $94.40 per person for study completion.

#### Intervention

Participants were each asked to complete 3 different types of dietary assessment:

*1*) FR via the ASA24 (Automated Self-Administered 24-hour Dietary Assessment Tool), version ASA24-US-2020 [17]: This validated, web-based tool enables multiple, automatically coded self-administered 24-h recalls and/or multiple day FRs. The National Cancer Institute (NCI) provides ASA24 to the research community at no cost. The ASA24 FR uses the USDA Food and Nutrient Database for Dietary Studies (FNDDS) as its nutrient database [18]. Completion time can range from 15 min to 30 min per day. For this study, we used the FR capabilities. Typically, a 3-d FR that includes 2 weekdays and 1 weekend day is acceptable as most representative of energy intake while being manageable from a completion and compliance standpoint.
*2*) FFQ via the DHQ (Dietary History Questionnaire) III [19]: This web-based assessment consists of 135 food and beverage line items and 26 dietary supplement questions to characterize habitual intake over the last 12 mo. The NCI provides the DHQ III to the research community at no cost. The DHQ III uses both FNDDS and Nutrition Data System for Research (NDSR) Food and Nutrient Database as its nutrient database [20,21]. Completion time can range from 30 min to 60 min.
*3*) DQPN: Through an image selection process, each participant finds the image that is most representative of his or her current dietary intake. The tool derives diet quality scores (1-to-10 scale) from the Healthy Eating Index (HEI) 2015 (0-to-100 scale), which is built using 13 components (food groups and nutrients) that correlate with the 2015–2020 Dietary Guidelines for Americans [22]. DQPN uses the NDSR Food and Nutrient Database. Completion time can range from 1 min to 4 min.

Between September and November 2021, participants were enrolled and asked to complete the diet assessments in the following sequence to minimize attrition and maximize time in between:

*Week 1:* DQPN plus 3-d FRs (2 weekdays and 1 weekend day)

*Week 2:* FFQ

*Week 3:* repeated DQPN and completion of any missed method, on separate days

Prior to study launch, a 1-d pilot was conducted to ensure participants could access, understand, and complete each task.

#### Data collection and statistical analysis

CloudResearch provided the participants with URL links and access codes as provided by the study authors but did not have access to participants’ assessment data. Demographic and other nondietary data collected via DQPN and FFQ included sex, age, physical activity level, height, and weight. Dietary data included macro- and micronutrient intake as well as dietary quality and food group components of the HEI score.

Descriptive statistics for diet quality (HEI), HEI food groups, and nutrient values were generated for each of the diet assessment methods. Correlations were generated via Pearson correlation. Energy adjustment by the nutrient density method was performed for the nutrient analysis. Test-retest reliability, or reproducibility, was calculated via Pearson correlation for DQPN’s measure of diet quality. In light of the multiple comparisons, we used a Bonferroni adjustment and considered a threshold of 0.004 as statistically significant. Notably, after seeing results of the above analyses (i.e., post-hoc), we also decided to evaluate food groups as estimated by DQPN against those estimated by the FFQ and FRs. Analyses were conducted using SAS version 9.4 (SAS Institute Inc).

#### Study approval

The study received an exempt determination (Pro00055341) by Advarra Institutional Review Board (www.advarra.com).

## Results

For the pilot, 15 participants were recruited, of whom 6 completed DQPN, 7 completed the DHQ, and 5 completed the ASA24, all without any difficulty. These results led to confidence that the technology and instructions were adequate to proceed with the full study.

For the full study, 90 participants were recruited, 79 completed the FFQ and DQPN, 61 completed the FRs and DQPN, and 58 completed all 3 dietary assessments. Baseline characteristics of the study participants and the diet pattern type and quality, as determined by DQPN, are shown in Table 1. Mean age (SD) of participants was 38 (11) y, and most were men (64%). Mean (SD) body mass index was 27 (6) kg/m^2^, and most participants reported low levels of physical activity (46%). The standard American diet was by far the most common dietary pattern (22 of 58 participants followed this pattern), and the overall quality of the diets was nearly at the midpoint of the HEI scale (59 on the 100-point scale).

**TABLE 1:** Characteristics, diet type, and diet quality of 58 study participants

Baseline characteristics	Category or unit	Mean (SD) or *n* (%)
Age	y	38 (11)
Sex	female	21(36)
BMI	kg/m^2^	27 (6)
Self-reported physical activity	Active	7 (12)
	Moderate	19 (32)
	Light	21 (36)
	Minimal	11 (19)
**Diet type**	**Mean HEI Score (SD)**	***n* (%)**
Standard American^1^	49 (26)	22 (38)
Flexitarian^2^	68 (21)	11 (19)
Southern-Style American^3^	62 (28)	7 (12)
Low-Fat^4^	56 (27)	6 (10)
Low-Carb^5^	64 (22)	3 (5)
Mediterranean^6^	60 (33)	3 (5)
No Red Meat (Standard American)^7^	83 (10)	3 (5)
Vegan^8^	63 (8)	2 (3)
Vegetarian^9^	76 (NA)	1 (2)
All	59 (25)	58

1 Standard American: Typically includes fruits, vegetables, grains, beans, nuts, seeds, dairy products, eggs, meats, poultry, and fish. May include highly processed foods, beverages, and ingredients.

2 Flexitarian: Mostly vegetarian diet that sometimes includes meat, fish, and/or poultry. May include some highly processed foods, beverages, and ingredients.

3 Southern-Style American: Reflective of the cuisine style to the Southern states of America, this diet includes fruits, vegetables, grains, beans, nuts, seeds, dairy products, eggs, meats, poultry, and fish. May include highly processed foods, beverages, and ingredients.

4 Low-Fat: Typically includes lean meats, poultry and fish, fruits and vegetables, grains, legumes, and low-fat dairy products. May include low-fat processed foods and limited amounts of nuts, seeds, nut butters, olives, avocado, cooking oils, fatty fish, and eggs.

5 Low-Carb: Typically includes lean meats, poultry, seafood, eggs, mostly non-starchy vegetables, whole fruits, nuts and seeds, and a variety of fats, with or without dairy/non-dairy products. Limits grains, legumes, and added sugars.

6 Mediterranean: Typically includes vegetables, fruits, nuts and seeds, whole grains, legumes, dairy products, seafood, and lean poultry. Emphasis on olive oil, herbs, spices, and red wine (moderation-optional).

7 No Red Meat: Variation of Standard American that excludes red meat.

8 Vegan: Comprised of 100% plant-based foods, including vegetables, fruits, grains, legumes, nuts, and seeds, and can also include soy products, oils, dairy substitutes, meat substitutes. Excludes all animal-based products.

9 Vegetarian: Typically includes vegetables, fruits, grains, legumes, nuts, seeds, dairy products, and eggs. Excludes all other animal-based products.

Descriptive statistics of diet quality, energy, and nutrients as well as Pearson correlations are shown in Table 2. The strongest correlations for DQPN were for diet quality; between DQPN and the FFQ, the correlation was 0.58 (*P* < 0.001), and between DQPN and FRs, the correlation was 0.56 (*P* < 0.001). The correlation between the FFQ and FRs for diet quality was slightly stronger (0.69, *P* < 0.0001). There was moderately strong and statistically significant correlation for fiber intake estimates between DQPN and FRs (0.39, *P =* 0.002) and DQPN and the FFQ (0.45, *P =* 0.0004). There appeared to be stronger correlations between the FFQ and FRs than between DQPN and these instruments. Energy adjustment did not materially change these results. The strongest correlations in the energy-adjustment models for DQPN were with fiber when compared to the FFQ (0.47, *P* = 0.0002).

**TABLE 2:** Diet quality and nutrient intake estimates with Pearson correlations from Diet Quality Photo Navigation, FRs, and FFQ^1^

	Nutrient values, unadjusted						Nutrient values, energy adjusted^2^					
	Mean (SD)			Pearson *r* (*P* value)^3^			Mean (SD)			Pearson *r* (*P* value)^3^		
	DQPN	FR	FFQ	DQPN vs. FR	DQPN vs. FFQ	FFQ vs. FR	DQPN	FR	FFQ	DQPN vs. FR	DQPN vs. FFQ	FFQ vs. FR
Diet quality (HEI-2015 score)	∗∗	∗∗	∗∗	∗∗	∗∗	∗∗	59 (25)	49 (11)	60 (11)	0.56 (<0.001)	0.58 (<0.001)	0.69 (<.0001)
Energy (kcal)	2400 (454)	1822 (744)	1819 (1083)	0.21 (0.11)	0.06 (0.65)	0.27 (0.0399)	∗∗	∗∗	∗∗	∗∗	∗∗	∗∗
Total sugar (g)	107.26 (25.35)	70.12 (43.97)	90.22 (64.44)	0.06 (0.63)	0.19 (0.15)	0.61 (<.0001)	90 (20)	82 (47)	103 (43)	0.31 (0.02)	0.18 (0.17)	0.60 (<.0001)
Added sugar (g)	57.80 (38.21)	10.32 (8.96)	60.68 (60.81)	0.23 (0.08)	0.26 (0.05)	0.63 (<.0001)	50 (35)	68 (43)	12 (10)	0.32 (0.01)	0.30 (0.02)	0.59 (<.0001)
Sodium (mg)	3695 (1127)	3210 (1552)	2926 (1353)	0.15 (0.26)	0.27 (0.04)	0.31 (0.0191)	3111 (839)	3697 (1489)	3301 (650)	0.11 (0.41)	-0.11 (0.41)	0.23 (0.0875)
Fiber (g)	28.76 (18.30)	15.03 (9.46)	16.47 (8.67)	0.39 (0.002)	0.45 (0.0004)	0.45 (0.0004)	23 (13)	17 (10)	19 (8)	0.40 (0.002)	0.47 (0.0002)	0.70 (<.0001)
Carbohydrates (g)	296 (77)	193 (87)	211 (118)	0.20 (0.12)	0.15 (0.26)	0.29 (0.0273)	245 (37)	218 (62)	234 (44)	0.16 (0.24)	0.29 (0.03)	0.58 (<.0001)
Protein (g)	98.58 (32.58)	76.64 (35.75)	74.44 (34.13)	0.17 (0.19)	0.16 (0.21)	0.30 (0.0228)	82 (21)	88 (33)	85 (18)	0.28 (0.03)	0.36 (0.006)	0.24 (0.0706)
Total fat (g)	94.49 (18.56)	76.65 (42.98)	66.81 (29.00)	0.05 (0.70)	0.16 (0.22)	0.40 (0.0017)	80 (13)	82 (25)	77 (16)	0.20 (0.13)	0.20 (0.14)	0.45 (0.0004)
Monounsaturated fat (g)	35.86 (8.85)	27.23 (17.25)	24.42 (10.73)	0.07 (0.58)	0.16 (0.21)	0.35 (0.0064)	30 (7)	29 (11)	28 (7)	0.09 (0.49)	0.23 (0.09)	0.44 (0.0005)
Polyunsaturated fat (g)	24.46 (6.88)	17.89 (12.04)	14.13 (6.88)	0.08 (0.55)	0.08 (0.58)	0.31 (0.0197)	20 (4)	20 (9)	16 (4)	0.10 (0.45)	0.15 (0.26)	0.47 (0.0002)
Saturated fat (g)	25.97 (9.9)	26.39 (11.89)	22.32 (10.4)	0.17 (0.1991)	0.28 (0.0324)	0.49 (0.0001)	22 (9)	26 (1)	26 (7)	0.22 (0.09)	0.28 (0.04)	0.35 (0.0067)

∗∗ not applicable

1 DQPN, Diet Quality Photo Navigation; FFQ, food frequency questionnaire; FR, food records.

2 Energy adjustment by nutrient density approach generated by dividing nutrient intake by energy intake (kcal) and multiplying by 2000 kcal

3 *P* < 0.05, 2-tailed

Table 3 shows intake estimates of food groups used in the calculation of the HEI score. In general, DQPN generated larger estimates of intake than those generated by the FFQ or FRs. Slightly stronger correlations appeared between DQPN and FRs and the FFQ in relation to food groups than between DQPN and the other 2 instruments in relation to nutrients (Table 2). There was a slightly stronger correlation between DQPN and the FFQ than between DQPN and FRs, especially with regard to fruit (0.37, *P* = 0.004), whole grains (0.43, *P* = 0.001), and nuts and seeds (0.48, *P ≤* 0.001). With the exception of whole grains, there were consistently stronger correlations between the FFQ and FRs than between DQPN and either instrument.

**TABLE 3:** Food group intake estimates and Pearson correlation from Diet Quality Photo Navigation, FRs, and FFQ^1^

Food group	Mean (SD)			Pearson correlation (*P* value)		
	DQPN	FR	FFQ	DQPN vs. FR	DQPN vs. FFQ	FR vs. FFQ
Fruit (cup eq)^2^	0.83 (0.94)	0.38 (0.65)	0.83 (0.79)	0.22 (0.09)	0.37 (0.004)	0.65 (<0.001)
Vegetables (cup eq)^2^	2.51 (1.97)	1.21 (0.75)	1.51 (1.14)	0.27 (0.04)	0.27 (0.04)	0.36 (0.005)
Legumes (cups / cup eq)^2^	0.15 (0.28)	0.06 (0.14)	0.10 (0.16)	0.18 (0.18)	0.02 (0.87)	0.64 (<0.001)
Nuts and Seeds (oz)	1.17 (1.49)	0.99 (1.66)	1.10 (1.47)	0.41 (0.001)	0.48 (<0.001)	0.52 (<0.001)
Whole grains (oz eq)^2^	2.41 (2.90)	1.10 (1.08)	0.91 (0.73)	0.39 (0.002)	0.43 (<0.001)	0.38 (0.003)
Refined grains (oz eq)^2^	5.86 (3.00)	5.40 (3.32)	3.69 (2.20)	0.36 (0.005)	0.10 (0.46)	0.30 (0.02)
Eggs (oz)	0.80 (0.42)	0.51 (0.55)	0.49 (0.48)	0.16 (0.24)	-0.05 (0.68)	0.17 (0.19)
Dairy (cup eq)^2^	2.13 (1.13)	1.54 (0.97)	1.66 (1.08)	0.01 (0.95)	0.26 (0.05)	0.31 (0.02)
Meat and seafood (oz eq)	5.46 (3.01)	4.18 (2.79)	3.74 (2.47)	0.22 (0.09)	0.25 (0.06)	0.41 (0.001)

1 DQPN, Diet Quality Photo Navigation; FFQ, food frequency questionnaire; FR, food record.

2 Component of Healthy Eating Index-2015

The test-retest reliability analysis included 69 participants who had repeated DQPN assessments within 14 d (mean 8.6 d). The results for diet quality (scores range from 1 to 10 with the digital user-facing display) showed a correlation of 0.70 (*P* = <0.0001).

## Discussion

In this comparison of 3 assessments of dietary intake, DQPN correlated well with the 2 dietary assessment methods in prevailing use, the 3-d FRs and the semiquantitative FFQ, when estimating overall diet quality. DQPN’s dietary intake estimates correlated with the FFQ slightly better than with FRs. Strong reliability for repeat assessments of diet quality from DQPN was also observed.

Prior research has shown a moderate correlation between DQPN and the Block FFQ for diet quality as measured by the Healthy Eating Index 2010 (HEI-2010) [23] and Alternate Healthy Eating Index 2010 (AHEI-2010) [24]. Pearson correlations (HEI-2010, *r* = 0.50, *P* < 0.001; AHEI-2010, *r* = 0.52, *P* < 0.001) used HEI-2010 and AHEI-2010 continuous values, while Spearman correlations (HEI-2010, *r* = 0.31, *P* < 0.05; AHEI-2010, *r* = 0.54, *P* < 0.001) used HEI and AHEI quintiles [8]. The current study updates these analyses with HEI-2015 scoring and confirms DQPN’s ability to capture diet quality.

In a separate earlier study, nutrient intake estimated from DQPN was higher than that estimated by 24-h recall [7,17]. DQPN had also estimated a higher energy intake than the recall with the difference being most pronounced at lower reported energy intakes. It had been noted that overestimation of physical activity on DQPN through self-report may lead to erroneous energy intake estimation [7]. There were significant moderate Spearman rank correlations between the 2 instruments for estimates of carbohydrate (*r =* 0.30), cholesterol (*r =* 0.46), and vitamins B1 (*r =* 0.30), B12 (*r =* 0.32), and E (*r =* 0.31). HEI correlation was −0.32. In the current study, DQPN also showed higher energy intake compared with the other 2 methods as well as significant correlations for fiber with both FRs and the FFQ. That fiber is most strongly correlated may be due to the strong correlation between DQPN and the other methods in capturing fruit, whole grain, and nuts and seeds intake.

That there was not a stronger correlation between DQPN and the other measures in terms of nutrient estimation may not be surprising. Certain nutrients track well with circumscribed dietary patterns and measures of diet quality, whereas others do not, and consequently, one may anticipate strong correlations between key nutrients of both deficiency and excess that define patterns and quality indices. Although the current study did not evaluate all such nutrients, a prior study of DQPN observed moderately strong correlation with nutrients such as fiber (0.64, *P* = 0.0001), potassium (0.58, *P* = 0.0001), lutein and zeaxanthin (0.58, *P* = 0.0001), and total carotenoids (0.44, *P* = 0.003) [13]. It is not clear why the current study did not observe stronger correlations with sodium, added sugar, and saturated fat—3 components that help define dietary quality—but it may pertain to a tendency for individuals to underreport foods that are deemed ‘unhealthy’ [13].

In this study, the FFQ and FRs correlated more strongly with one another than either did with DQPN, which may be because these methods are both based on food-level capture rather than dietary pattern capture. Optimal performance of a new dietary assessment method may involve meaningful but limited correlation with the prevailing methods. Even biomarkers are imperfect correlates of dietary intake. They must be directly related to intake and not subject to homeostasis or substantial interindividual differences in metabolism, thus allowing for an estimate of intake without systematic bias [25]. Only a limited number of such recovery biomarkers are currently known, including doubly labeled water for energy intake, urinary nitrogen for protein intake, urinary potassium for potassium intake, and urinary sodium for sodium intake [25]. To better understand discrepancies among dietary intake assessment methods would require the entirely reliable reference standard offered by a metabolic ward. Although costly and labor-intensive, metabolic ward research in this area is warranted.

The sources of error in diet assessment instruments influence correlation coefficients, and thus, a key step in validation studies is limiting correlated errors. Random within-person error in the measurement of one or both dietary variables tends to reduce correlation coefficients toward zero (attenuation), whereas systematic between-person errors (in which all subjects are measured too high or too low by same amount) do not affect correlation coefficients [26].

Importantly, error in DQPN is likely different than that seen in FRs and FFQs. Random within-person error on DQPN may be limited given that daily intake is not assessed but rather habitual intake. With FRs, random error is expected to be largely due to day-to-day diet variation, which is not true error but rather deviation from mean habitual intake. Random error in DQPN may be a result of the diet map not having a photographic “perfect fit” for every user of the tool. Additionally, a user may choose the “closest” fit which may be very different for some but less so for others. Some users may also choose or reject a dietary image based on strong feelings about a given food or ingredient and as a result misrepresent his or her overall dietary pattern or quality. With repeat measurements, such within-person error should be expected to estimate the correct mean intake but may have wide variation [26].

Systematic error (bias) in DQPN would be due to inaccurate reporting associated with the potential cognitive challenge of understanding what DQPN is requesting of the user (participant), as well as features of the instrument, such as the finite image library and the limited detail about foods in the images [25]. This type of error may affect estimates of mean and variation for foods and nutrients [26]. FRs, by contrast, may have missing foods and beverages that the participant does not wish to reveal, as well as errors related to changes in diet resulting from the act of recording and those due to the uneven quality of reporting across participants, all of which may contribute to systematic error [25]. Low levels of participant literacy and/or numeracy may present systematic error with traditional diet assessment instruments. DQPN’s image pattern approach does not depend on a high degree of literacy or numeracy; additionally, it would be expected that any cognitive challenges its digital interface presents would be different than those seen with FRs or FFQs. A shared nutrient database, however, may present a correlated systematic error.

With regard to energy adjustment, correlations between 2 dietary assessment instruments are typically higher using energy-adjusted nutrients than for non–energy-adjusted nutrients, and this was seen in the present study [27]. Energy adjustment assumes that individuals misreport intake of foods and beverages to a similar degree and in the same direction [27]. Although this assumption does not hold up all the time, and less healthy foods tend to be under-reported to a greater degree than more healthy foods, evidence suggests that the assumption is reasonable [27].

There are important strengths and limitations of the present study to consider. The study enrolled more than its target number of participants and thus was well powered to identify differences between dietary assessments. Inclusion of study participants without recent dietary changes and who could complete the assessments in close temporal proximity to each other allowed for capture of dietary data with little risk of diet changes impacting either short-term or long-term intake. The HEI-2015 used in this study has recently been reviewed and renamed to HEI-2020 to align with the USDA’s 2020-2025 guidelines and therefore, is the most up-to-date dietary quality index [[28], [29], [30]]. In addition, as the average HEI score for Americans is 58 of 100, our study population lends itself to good external generalizability [28]. The online distribution of the assessments allowed for blinding and minimized potential researcher influence. However, photographs of foods used in DQPN may differ from foods described in the FFQ and FRs, thus affecting respondents’ estimation of portion size and intake. Portion size for different foods contributing to the DQPN dietary pattern and intake level identified is predicated on proportional representation of the various food groups in the composite image. This eliminates the need to rely on specific recall of typical serving sizes by food group or ingredient. We cannot exclude the possibility that participants misreported their dietary intake to appear healthier than it actually was; however, one could anticipate that if such occurred it would occur similarly on all 3 assessments and thus may not impact correlations.

In conclusion, the current study offers evidence that DQPN is comparable to traditional diet assessment tools for estimating overall diet quality. In addition, the new tool shows good test-retest reliability for estimating diet quality, providing further support for its performance as a diet assessment tool. Its performance and scalability may recommend it in urgently needed efforts to make dietary intake assessment a universal part of clinical care.

## Author contributions

The authors’ responsibilities were as follows—DLK, ABM, LQR: designed research; LQR: conducted research; VLN: analyzed data; ABM: wrote the paper; DLK, VLN, LQR: reviewed and edited the paper; and all authors: read and approved the final manuscript.

## Conflict of interest

DLK is CEO and a shareholder of Diet ID, Inc. AMB and LQR are option holders in Diet ID, Inc. VYN holds no financial interest in Diet ID, Inc.

## Funding

The study was funded by Diet ID, Inc.

## Data availability

Data described in the manuscript will be made available upon request by qualified scientists for appropriate uses.

## Declaration of interests

The authors declare the following financial interests/personal relationships which may be considered as potential competing interests: David L. Katz reports a relationship with Diet ID, Inc that includes: employment and equity or stocks. Adam M. Bernstein reports a relationship with Diet ID, Inc that includes: consulting or advisory and equity or stocks. Lauren Q. Rhee reports a relationship with Diet ID, Inc that includes: employment and equity or stocks. Valentine Y. Njike reports a relationship with Diet ID, Inc that includes: consulting or advisory. David L. Katz has patent Diet mapping processes and systems to optimize diet quality and/or minimize environmental impact issued to Diet ID, Inc. Lauren Q. Rhee has patent Diet mapping processes and systems to optimize diet quality and/or minimize environmental impact issued to Diet ID, Inc.
